# Comprehensive characterization of the WRKY gene family and their potential roles in regulation phenylphenalenone biosynthesis in *Musella lasiocarpa*

**DOI:** 10.3389/fpls.2025.1570758

**Published:** 2025-03-12

**Authors:** Long Huang, Pirui Li, Mei Tian, Xu Feng, Yu Chen, Boya Feng, Wanli Zhao

**Affiliations:** Jiangsu Key Laboratory for the Research and Utilization of Plant Resources, Jiangsu Province Engineering Research Center of Eco-cultivation and High-value Utilization of Chinese Medicinal Materials, Institute of Botany, Jiangsu Province and Chinese Academy of Sciences (Nanjing Botanical Garden Mem. Sun Yat-Sen), Nanjing, China

**Keywords:** WRKY, *Musella lasiocarpa*, regulation, phenylphenalenone biosynthesis, O-methyl transferase

## Abstract

Phenylphenalenone is an important phytoalexin for banana plant protection, yet the mechanisms governing its biosynthesis and regulation remain unclear in plant. WRKY transcription factors play essential roles in modulating plant growth, development, and the biosynthesis of secondary metabolites. In this study, we identified 158 WRKY genes (*MlWRKYs*) from a phenylphenalenone-rich plant species *Musella lasiocarpa*. Phylogenetic analysis classified the *MlWRKY* genes into three distinct subfamilies: type I, type II, and type III. Chromosomal distribution revealed that the *MlWRKY* genes are clustered on nine respective chromosomes. Additionally, synteny analysis between *M. lasiocarpa* and *Musa balbisiana* uncovered highly conserved collinear regions. *MIWRKY15*, *MIWRKY111*, *MIWRKY122* were identified as candidate genes for regulating PhPNs biosynthesis by integration of multi-omics approaches. We further investigated the expression pattern of *MIWRKY15*, *MIWRKY111*, *MIWRKY122* genes, as well as their putative target genes *MlOMT22* and *MlOMT27*, the known phenylphenalenone biosynthesis genes in various tissues, including leaves, stems, roots, and seeds. *MlWRKY15* and *MlOMT22* showed similar expression patterns across tissues. *MlWRKY122* and *MlOMT27* also displayed consistent expression patterns, suggesting *MlWRKY122* may regulate *MlOMT27*. Additionally, *MlWRKY111*’s expression was inversely correlated with *MlOMT27*, indicating a potential negative regulation of *MlOMT27* by *MlWRKY111*. This study provides valuable insights into the WRKY family in *M. lasiocarpa* and will serve as a useful genetic resource for elucidating the regulatory mechanisms of phenylphenalenone biosynthesis.

## 1 Introduction

Phenylphenalenones (PhPNs) are predominantly found in monocot families, such as Strelitziaceae and Musaceae (Norman et al., 2019). These compounds have been identified as significant phytoalexins in wild banana species, making them a valuable resource for developing disease-resistant banana varieties (Flors and Nonell, 2006; Chen et al., 2018). However, cultivated bananas typically exhibit low concentrations and limited structural diversity of PhPNs. Genetic modification techniques represent a promising strategy to address this issue through the targeted manipulation of biosynthetic pathways and enzymes associated with PhPN production, thereby conferring enhanced disease resistance in banana plants. Research has indicated that PhPNs are synthesized via the phenylpropanoid biosynthetic pathway, with their linear precursors undergoing intramolecular cyclization (Norman et al., 2019). For example, a chalcone synthase named WtPKS1, which catalyzes the initial step in diarylheptanoid biosynthesis, was characterized from *Wachendorfia thyrsiflora* (Brand et al., 2006). Our research group has previously characterized three *O*-methyltransferases (OMT) involved in the phenylphenalenone biosynthetic pathway from Chinese dwarf banana *Musella lasiocarpa* (Zhao et al., 2024). However, the mechanisms governing PhPN biosynthesis and regulation remain unknown. *M. lasiocarpa*, an endemic species in China and the sole representative of the genus Musella, is primarily distributed in southwestern regions, particularly in Yunnan Province (Liu et al., 2003; Ma et al., 2019). Traditionally, the flowers and bracts of this plant have been utilized in folk medicine for their hemostatic and anti-inflammatory properties (Liu et al., 2003). Recent phytochemical studies have revealed that *M. lasiocarpa* contains a variety of PhPNs as well as linear diarylheptanoids, which are believed to be precursors in the biosynthesis of PhPNs (Dong et al., 2011). Consequently, *M. lasiocarpa* exhibits considerable potential as a model organism for elucidating the biosynthetic pathways of PhPN.

WRKY transcription factors constitute a vital class of plant genes that play an indispensable role in various physiological processes, including stress responses and the biosynthesis of secondary metabolites (Jiang et al., 2017; Viana et al., 2018; Liu et al., 2020; Wang et al., 2022; Zhang et al., 2023; Ma et al., 2024b). These transcription factors regulate the expression of target genes by specifically binding to W-box cis-elements [(T)TGAC(C/T)] in promoter regions. Additionally, they interact with a diverse array of proteins to execute their functions across multiple signaling pathways (Chen et al., 2017; Javed and Gao, 2023). WRKY transcription factors orchestrate the biosynthesis of plant secondary metabolites through transcriptional regulation of rate-limiting enzymes in secondary metabolic pathways (Schluttenhofer and Yuan, 2015). PeWRKY30, a crucial transcription factor that is co-expressed alongside flavonoid accumulation in yellow-fruited *Passiflora edulis*, may contribute to enhancing resistance against both biotic and abiotic stresses (Ma et al., 2024a). The PsWRKY transcription factor in *Papaver somniferum* binds to the W-box in the promoter regions of benzylisoquinoline alkaloid pathway genes, thus stimulating transcriptional activity from the tyrosine/DOPA decarboxylase promoter (Mishra et al., 2013). Several members of the WRKY family play pivotal roles in regulating terpenoid biosynthesis pathways. Specifically, the *Artemisia annua* transcription factor *AaWRKY1* enhances *ADS* expression by directly binding to the promoter region of the artemisinin biosynthesis gene *ADS*, thereby modulating artemisinin biosynthesis (Han et al., 2014; Jiang et al., 2016). Similarly, overexpression of *TcWRKY8* and *TcWRKY47* in *Taxus chinensis* significantly increases the expression levels of paclitaxel-related synthase genes, thereby enhancing the biosynthetic pathway of paclitaxel (Han et al., 2014; Jiang et al., 2016). Similarly, overexpression of *TcWRKY8* and *TcWRKY47* in *Taxus chinensis* significantly upregulates the expression levels of paclitaxel-related synthase genes, leading to enhanced biosynthesis of paclitaxel (Zhang et al., 2018). Furthermore, the homodimer of GhWRKY41 from *Gossypium hirsutum* directly enhances the expression of *GhC4H* and *Gh4CL*, which in turn regulates the accumulation of lignin and flavonoids (Xiao et al., 2023). To our konwledge, the regulatory role of WRKY transcription factors in PhPN biosynthesis remains unexplored, highlighting a critical gap in our understanding of this metabolic pathway.

In this study, we aimed to comprehensively characterize the WRKY gene family in *M. lasiocarpa* through genome-wide identification and analysis, focusing on gene classification, chromosomal distribution, and phylogenetic relationships, to provide a foundation for future functional studies. Through integration of multi-omics approaches, *MIWRKY15*, *MIWRKY111*, and *MIWRKY122* were identified as candidate genes potentially involved in regulating PhPNs biosynthesis. These findings will provide a robust foundation for molecular studies and genetic engineering initiatives aimed at enhancing disease resistance in Musaceae.

## 2 Materials and methods

### 2.1 Plant material

The *M. lasiocarpa* was gathered from Nanhua County, Yunnan Province, China (coordinates: 118°50’38”E, 32°3’44”N) and subsequently relocated to an experimental field in Nanjing, China (coordinates: 101°1’2”E, 25°9’54”N). Species identification of the experimental materials was conducted by Professor Yu Chen from the Institute of Botany Jiangsu Province, Chinese Academy of Sciences. For total RNA extraction experiments, leaf, stem, and seed samples were harvested from the same plant individual at three developmental stages (yellow seed, S2; brown seed, S4; and black seed, S6).

### 2.2 Identification of MlWRKY genes from *M. lasiocarpa* genome

The genome sequence of *M. lasiocarpa* (GenBank number: PRJNA1009687) was retrieved from our research group. The AtWRKY protein sequences were retrieved from the Arabidopsis Information Resource database available at https://www.arabidopsis.org. Potential MlWRKY proteins were initially identified through homology searches using BLAST and Hidden Markov Model (HMM) algorithms. The predicted MlWRKY annotations were then validated by cross-referencing with the Swiss-Prot database, followed by further analysis using the NCBI Conserved Domain Database (CDD) at https://www.ncbi.nlm.nih.gov/cdd. The presence of conserved domains in these candidate proteins was verified using the Pfam database. Various physicochemical properties, including coding sequence (CDS) length, isoelectric point, and molecular weight, were predicted for the identified MlWRKY proteins using the EXPASy-ProtParam tool.

### 2.3 Chromosomal localization, phylogenetic analysis, and collinearity analysis

Phylogenetic analysis was performed using the Maximum Likelihood approach, supported by 1,000 bootstrap replicates, utilizing MEGA version 5.05. This analysis utilized the Jones-Taylor-Thornton substitution model with a stringent requirement of minimum 95% site coverage. Conserved domains were identified through Batch-Search and TBtools software (Chen et al., 2020). To better understand the functional characteristics of MlWRKY proteins, their conserved domains were analyzed via the MEME program (https://meme-suite.org/meme/). Furthermore, TBtools was used to create a distribution map that visualizes the organization of *MlWRKY* genes. The Dual Synteny Plotter feature in TBtools was employed to analyze the syntenic relationships between MlWRKYs and WRKY genes from various species. Genomic data for collinearity analysis were obtained from the following sources: *Arabidopsis thaliana* (https://www.arabidopsis.org), *Oryza sativa* (https://plants.ensembl.org/info/data/ftp/index.html), and *Musa balbisiana* (https://www.ncbi.nlm.nih.gov/datasets/genome/). To investigate the tissue-specific expression patterns of *MlWRKYs* in *M. lasiocarpa*, we utilized an RNA-seq dataset (accession number PRJNA100968) deposited by our team in NCBI.

### 2.4 RNA extraction and quantitative real-time PCR

To isolate total RNA, we employed the FastPure Universal Plant Total RNA Isolation Kit (RC411) from Vazyme Biotech Co., Ltd. (Nanjing, China), following the protocol specified by the manufacturer. For qRT-PCR purposes, the HiScript III 1st Strand cDNA Synthesis Kit (Vazyme) was used to synthesize cDNA from the extracted total RNA. The qRT-PCR experiments were performed on the qTOWER 2.2 system, manufactured by Analytik Jena in Germany. The PCR amplification conditions consisted of 40 cycles with denaturation at 95°C for 10 seconds and annealing/extension at 60°C for 30 seconds. To assess relative expression levels, the *EF-α* gene from *M. lasiocarpa* served as an internal control, and the comparative cycle threshold (2^−ΔΔCt^) method was applied, incorporating *t* values for analysis. Each sample included both biological and technical replicates. Primer sequences for all target transcripts were designed using Primer Premier software (**Supplementary Table S1**).

### 2.5 Statistical analyses and data visualization

To demonstrate the significant differences between the two groups, a two-tailed unpaired Student’s t-test was employed. The thresholds for significance were established as *p < 0.05, **p < 0.01, and ***p < 0.001. Results are presented as mean ± SD. All statistical analyses were conducted using GraphPad Prism 9. Additionally, TBtools was utilized to generate a distribution map that illustrates the arrangement of MlWRKY genes.

## 3 Results

### 3.1 Genome-wide identification of *MlWRKY* gene Family Members in *M. lasiocarpa*

We identified *MlWRKY* genes in the *M. lasiocarpa* genome by screening for conserved WRKY domains, followed by manual validation of gene structures and functional motifs. A total of 158 MlWRKY proteins, each containing more than 100 amino acids, were identified after filtering out incomplete sequences and shorter variants. The 158 genes were designated as MlWRKY1 to MlWRKY158 according to their chromosomal localization (**Table 1**). To better understand the molecular characteristics of these MlWRKY members, their physiological and biochemical properties were examined. The proteins varied in length from 102 to 2004 amino acids (aa), with corresponding molecular weights (MW) between 11,294.9 and 223,829.29 Daltons. Their isoelectric points also differed, ranging from 4.41 to 10.45. Eighteen distinct domains were identified through the analysis of conserved motifs using the MEME program. The results demonstrated that all *MlWRKY* genes contain at least one WRKY conserved domain, thereby highlighting the conservation these genes (**Supplementary Figure S1**).

**Table 1:** Characteristics of MlWRKY transcription factors.

Name	Gene IDs	Locus	NO. (aa)	MW (Da)	PI	Name	Gene IDs	Locus	NO. (aa)	MW (Da)	PI
MlWRKY1	Ml01G0317	Chr1	473	52273.10	5.47	MlWRKY80	Ml06G0788	Chr6	260	28525.38	9.11
MlWRKY2	Ml01G0536	Chr1	302	32340.62	9.79	MlWRKY81	Ml06G1278	Chr6	548	58666.24	6.42
MlWRKY3	Ml01G0666	Chr1	365	40401.10	9.62	MlWRKY82	Ml06G1699	Chr6	413	44435.04	5.42
MlWRKY4	Ml01G0710	Chr1	626	67246.70	5.84	MlWRKY83	Ml06G1744	Chr6	733	79177.78	5.83
MlWRKY5	Ml01G0771	Chr1	506	54573.69	8.10	MlWRKY84	Ml06G1822	Chr6	102	11316.93	9.73
MlWRKY6	Ml01G0884	Chr1	327	35910.83	6.06	MlWRKY85	Ml06G1973	Chr6	215	23319.38	9.75
MlWRKY7	Ml01G0936	Chr1	286	30524.66	5.10	MlWRKY86	Ml06G2093	Chr6	360	38686.99	5.65
MlWRKY8	Ml01G0944	Chr1	172	19669.32	8.83	MlWRKY87	Ml06G2183	Chr6	271	30192.70	8.50
MlWRKY9	Ml01G1014	Chr1	183	21349.96	8.39	MlWRKY88	Ml06G2274	Chr6	397	43714.91	4.91
MlWRKY10	Ml01G1204	Chr1	331	37010.46	6.11	MlWRKY89	Ml06G2303	Chr6	470	50456.68	6.68
MlWRKY11	Ml01G1205	Chr1	310	34168.42	5.85	MlWRKY90	Ml07G0134	Chr7	102	11294.90	10.05
MlWRKY12	Ml01G1460	Chr1	314	35078.50	8.14	MlWRKY91	Ml07G0219	Chr7	499	53794.85	5.68
MlWRKY13	Ml01G1888	Chr1	159	18567.89	9.38	MlWRKY92	Ml07G0264	Chr7	184	20973.53	8.81
MlWRKY14	Ml01G2199	Chr1	556	59147.67	5.93	MlWRKY93	Ml07G0394	Chr7	537	57679.55	6.63
MlWRKY15	Ml01G2683	Chr1	340	37892.85	9.81	MlWRKY94	Ml07G0962	Chr7	596	64197.00	6.44
MlWRKY16	Ml01G3005	Chr1	189	22239.99	7.09	MlWRKY95	Ml07G1183	Chr7	291	31461.39	6.51
MlWRKY17	Ml01G3046	Chr1	305	33311.84	7.70	MlWRKY96	Ml07G1264	Chr7	540	57825.64	6.15
MlWRKY18	Ml01G3154	Chr1	348	37628.82	6.08	MlWRKY97	Ml07G1552	Chr7	342	37962.41	7.14
MlWRKY19	Ml01G3207	Chr1	488	52620.92	6.28	MlWRKY98	Ml07G1729	Chr7	324	34988.17	10.06
MlWRKY20	Ml01G3365	Chr1	387	42393.16	5.13	MlWRKY99	Ml07G1758	Chr7	718	77997.51	5.76
MlWRKY21	Ml01G3504	Chr1	209	23320.17	9.33	MlWRKY100	Ml07G1867	Chr7	355	39518.03	5.73
MlWRKY22	Ml02G0061	Chr2	498	54093.42	5.73	MlWRKY101	Ml07G2182	Chr7	512	56232.58	6.21
MlWRKY23	Ml02G0497	Chr2	329	35800.34	6.41	MlWRKY102	Ml07G2194	Chr7	143	16062.31	10.15
MlWRKY24	Ml02G1305	Chr2	287	31799.81	6.75	MlWRKY103	Ml07G2256	Chr7	732	79427.83	5.43
MlWRKY25	Ml02G1344	Chr2	520	56358.34	7.60	MlWRKY104	Ml07G2422	Chr7	311	34127.08	4.99
MlWRKY26	Ml02G1349	Chr2	304	33080.84	6.00	MlWRKY105	Ml07G2632	Chr7	282	30239.98	9.08
MlWRKY27	Ml02G1642	Chr2	687	75628.83	9.74	MlWRKY106	Ml07G2633	Chr7	549	58957.28	7.59
MlWRKY28	Ml02G2184	Chr2	282	30653.46	9.04	MlWRKY107	Ml07G2864	Chr7	337	37569.61	9.74
MlWRKY29	Ml02G2611	Chr2	411	45111.18	5.60	MlWRKY108	Ml07G3051	Chr7	539	57073.73	6.82
MlWRKY30	Ml02G3094	Chr2	567	61468.45	6.58	MlWRKY109	Ml07G3406	Chr7	563	60198.76	6.29
MlWRKY31	Ml02G3205	Chr2	354	39334.04	8.31	MlWRKY110	Ml07G4047	Chr7	310	33244.29	6.16
MlWRKY32	Ml02G3319	Chr2	346	38377.51	9.55	MlWRKY111	Ml07G4183	Chr7	325	35520.62	9.44
MlWRKY33	Ml02G3398	Chr2	635	68693.87	9.85	MlWRKY112	Ml07G4231	Chr7	257	28564.27	8.49
MlWRKY34	Ml02G3584	Chr2	549	58310.33	6.35	MlWRKY113	Ml07G4401	Chr7	564	60568.19	6.60
MlWRKY35	Ml03G0328	Chr3	340	37911.06	10.14	MlWRKY114	Ml07G4518	Chr7	291	32511.10	9.77
MlWRKY36	Ml03G0950	Chr3	269	29879.73	6.59	MlWRKY115	Ml07G4606	Chr7	490	52406.22	6.28
MlWRKY37	Ml03G1400	Chr3	325	34925.47	5.35	MlWRKY116	Ml07G4784	Chr7	461	50161.46	5.44
MlWRKY38	Ml03G1470	Chr3	196	22092.74	8.25	MlWRKY117	Ml08G0383	Chr8	199	21156.88	4.41
MlWRKY39	Ml03G2257	Chr3	323	35574.28	6.08	MlWRKY118	Ml08G0449	Chr8	357	38512.56	5.70
MlWRKY40	Ml03G2290	Chr3	2004	223829.29	8.90	MlWRKY119	Ml08G0470	Chr8	560	59862.16	7.68
MlWRKY41	Ml03G2399	Chr3	612	66022.42	6.32	MlWRKY120	Ml08G0553	Chr8	330	36140.44	5.66
MlWRKY42	Ml03G2559	Chr3	275	30579.94	9.86	MlWRKY121	Ml08G0703	Chr8	284	29828.90	5.35
MlWRKY43	Ml03G2843	Chr3	181	19560.37	4.70	MlWRKY122	Ml08G0919	Chr8	728	79329.94	5.53
MlWRKY44	Ml03G3295	Chr3	210	22954.47	6.30	MlWRKY123	Ml08G1080	Chr8	324	36233.18	7.57
MlWRKY45	Ml03G3311	Chr3	514	56876.27	5.08	MlWRKY124	Ml08G1164	Chr8	248	27711.12	8.38
MlWRKY46	Ml03G3672	Chr3	300	33246.26	8.37	MlWRKY125	Ml08G1362	Chr8	202	22727.59	9.01
MlWRKY47	Ml03G3685	Chr3	219	23521.40	6.22	MlWRKY126	Ml08G1471	Chr8	425	46817.58	8.54
MlWRKY48	Ml03G3721	Chr3	289	31609.70	7.24	MlWRKY127	Ml08G1606	Chr8	492	52396.82	6.21
MlWRKY49	Ml03G3836	Chr3	441	48672.21	8.51	MlWRKY128	Ml08G1609	Chr8	273	30177.87	6.67
MlWRKY50	Ml04G0010	Chr4	308	33694.60	5.22	MlWRKY129	Ml08G1920	Chr8	387	42126.78	7.57
MlWRKY51	Ml04G0016	Chr4	225	24648.92	9.01	MlWRKY130	Ml08G2377	Chr8	306	33133.10	9.70
MlWRKY52	Ml04G0060	Chr4	382	41538.43	5.87	MlWRKY131	Ml08G2606	Chr8	334	35388.14	8.22
MlWRKY53	Ml04G0751	Chr4	326	34495.31	9.65	MlWRKY132	Ml08G2872	Chr8	289	30643.84	10.23
MlWRKY54	Ml04G0926	Chr4	221	24964.33	10.05	MlWRKY133	Ml09G0049	Chr9	338	37295.79	6.31
MlWRKY55	Ml04G1073	Chr4	316	35723.39	6.27	MlWRKY134	Ml09G0061	Chr9	755	81270.90	6.69
MlWRKY56	Ml04G1074	Chr4	254	29323.00	6.26	MlWRKY135	Ml09G0105	Chr9	274	30248.87	6.95
MlWRKY57	Ml04G1075	Chr4	290	32576.57	5.49	MlWRKY136	Ml09G0188	Chr9	522	55540.26	6.90
MlWRKY58	Ml04G1076	Chr4	297	33556.54	5.21	MlWRKY137	Ml09G0213	Chr9	683	73918.18	5.83
MlWRKY59	Ml04G1263	Chr4	309	34371.21	5.63	MlWRKY138	Ml09G0670	Chr9	263	29721.38	6.82
MlWRKY60	Ml04G1577	Chr4	299	33286.66	6.43	MlWRKY139	Ml09G0719	Chr9	168	19924.84	10.45
MlWRKY61	Ml04G1756	Chr4	255	28787.15	5.88	MlWRKY140	Ml09G0776	Chr9	280	29909.99	5.54
MlWRKY62	Ml04G2131	Chr4	453	49608.83	9.32	MlWRKY141	Ml09G0811	Chr9	518	56161.66	9.15
MlWRKY63	Ml04G3053	Chr4	359	39670.01	9.10	MlWRKY142	Ml09G0900	Chr9	521	55607.36	6.38
MlWRKY64	Ml05G0583	Chr5	168	18565.24	4.77	MlWRKY143	Ml09G1249	Chr9	304	33502.37	10.00
MlWRKY65	Ml05G0584	Chr5	230	24476.78	9.54	MlWRKY144	Ml09G1357	Chr9	329	36297.54	6.89
MlWRKY66	Ml05G0962	Chr5	252	27734.09	8.46	MlWRKY145	Ml09G1487	Chr9	285	31599.44	6.87
MlWRKY67	Ml05G1149	Chr5	113	12933.52	9.00	MlWRKY146	Ml09G1883	Chr9	272	30141.01	7.68
MlWRKY68	Ml05G1219	Chr5	309	33256.72	9.36	MlWRKY147	Ml09G1923	Chr9	319	34778.72	9.73
MlWRKY69	Ml05G1441	Chr5	376	40599.97	8.87	MlWRKY148	Ml09G2009	Chr9	280	31302.72	4.78
MlWRKY70	Ml05G1464	Chr5	288	31678.47	6.32	MlWRKY149	Ml09G2092	Chr9	363	40184.56	5.87
MlWRKY71	Ml05G1489	Chr5	299	33433.19	7.71	MlWRKY150	Ml09G2161	Chr9	588	63709.70	6.20
MlWRKY72	Ml05G2531	Chr5	283	31728.48	5.46	MlWRKY151	Ml09G2167	Chr9	238	26509.80	9.21
MlWRKY73	Ml05G2908	Chr5	481	52309.35	7.26	MlWRKY152	Ml09G2523	Chr9	139	15320.15	5.34
MlWRKY74	Ml05G3064	Chr5	368	40785.96	7.10	MlWRKY153	Ml09G2600	Chr9	570	60722.21	5.61
MlWRKY75	Ml05G3094	Chr5	438	47031.14	8.66	MlWRKY154	Ml09G2773	Chr9	189	21172.72	8.73
MlWRKY76	Ml05G3852	Chr5	249	27432.84	8.59	MlWRKY155	Ml09G2943	Chr9	314	34289.84	9.04
MlWRKY77	Ml05G4030	Chr5	377	40398.81	5.18	MlWRKY156	Ml09G3314	Chr9	123	14251.94	8.89
MlWRKY78	Ml06G0572	Chr6	733	79569.36	5.62	MlWRKY157	Ml09G3410	Chr9	512	56121.01	8.84
MlWRKY79	Ml06G0764	Chr6	305	33186.00	9.99	MlWRKY158	Ml09G3414	Chr9	259	28178.58	7.12

### 3.2 Chromosomal localization of *MlWRKYs* genes

**Figure 1:**
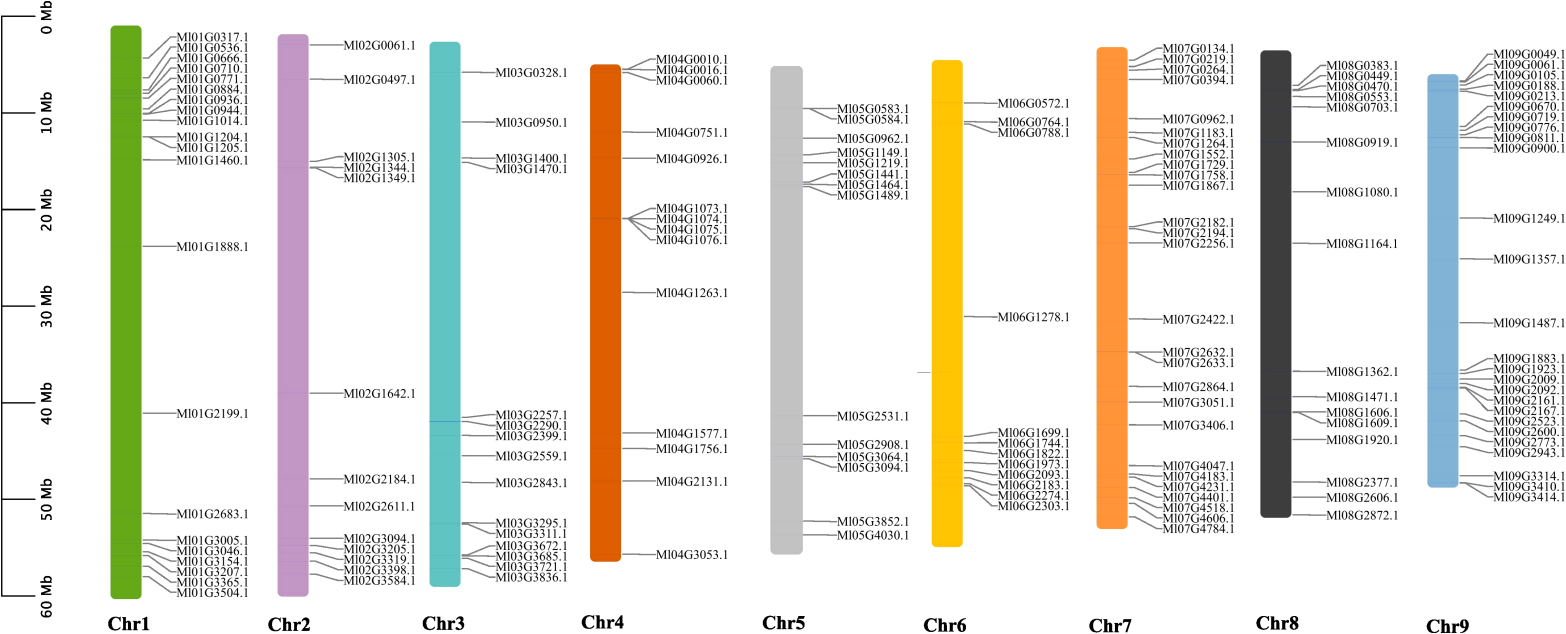
Chromosomal distribution of *MlWRKY* genes within the *M. lasiocarpa* genome. A total of 158 *MlWRKY* genes were localized across nine chromosomes in *M. lasiocarpa*. The left side of the figure indicates the scale in megabases (Mb). Each chromosome is depicted as a vertical line, with its name labeled beneath the corresponding line.

A chromosomal mapping approach was developed to investigate the genetic variation and duplication events within the WRKY family in *M. lasiocarpa*. 158 *MlWRKY* genes were unevenly distributed across nine *M. lasiocarpa* chromosomes (as shown in **Figure 1**). Chromosome 7 (chr7) harbored the highest number of *MlWRKY* genes (27), followed by chr9 (26), chr1 (21), chr8 (16), chr3 (15), chr4 (14), chr5 (14), and chr2 (13). Whereas only 12 genes were found on chr6.

### 3.3 Phylogenetic and Synteny analysis of MlWRKYs

**Figure 2:**
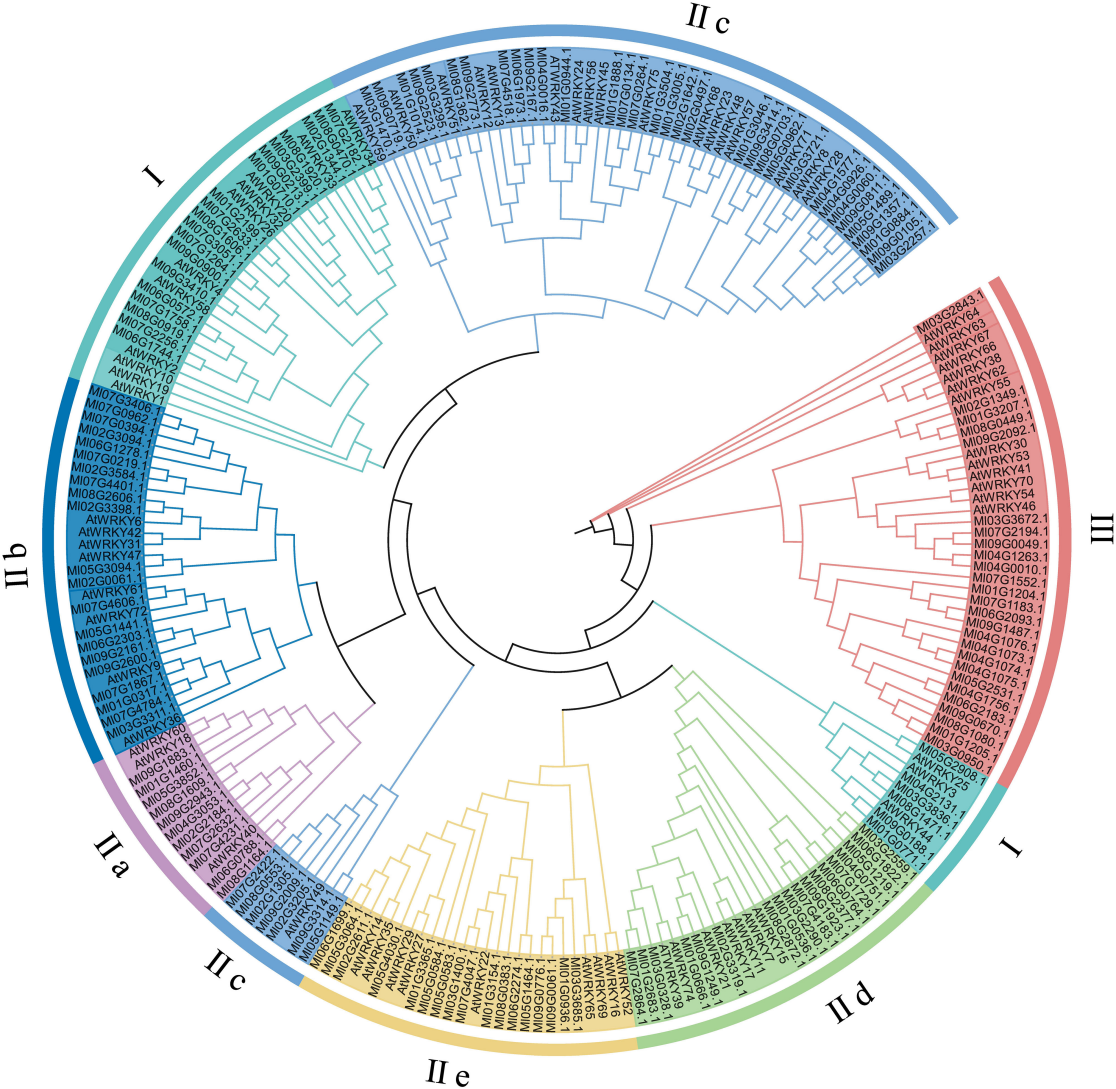
Phylogenetic relationships of WRKY gene families in *M. lasiocarpa* and *A. thaliana* were reconstructed using the maximum likelihood (ML) method in MEGA5. Multiple sequence alignment was performed with MUSCLE, and the best-fit substitution model (JTT+G) was selected. Branch support was evaluated with 1000 bootstrap replicates.

To examine the evolutionary relationship between MlWRKYs in *M. lasiocarpa* and the *A. thaliana*, a maximum likelihood phylogenetic tree was constructed. The MlWRKY family was classified into three major groups (Types I–III), in accordance with the classification of AtWRKYs from *A. thaliana* (**Figure 2**). In *M. lasiocarpa*, Type I included 25 members, compared to 14 in *A. thaliana*. Type II represented the largest group, with 116 MlWRKY proteins, and was subdivided into five subgroups: IIa, IIb, IIc, IId, and IIe. Furthermore, Type III contained 17 MlWRKY members, exceeding the 13 found in *A. thaliana*.

**Figure 3:**
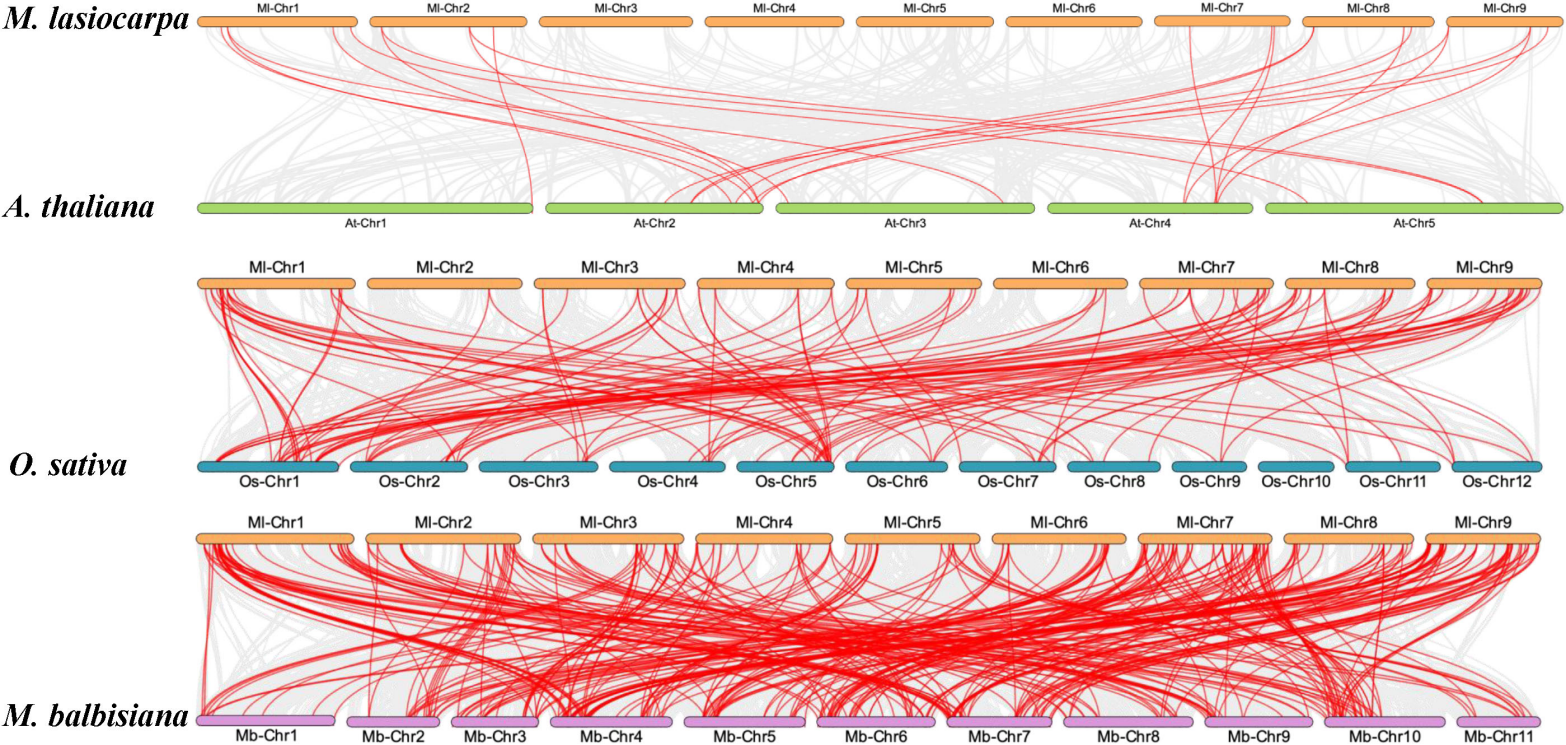
The collinearity gene pair analysis of WRKY among *M. lasiocarpa* (Ml), *M. balbisiana* (Mb), *A. thaliana*, (At) and *O. sativa* (Os). The *M. lasiocarpa*, *A. thaliana*, *O. sativa*, and *M. balbisiana* were represented by saffron yellow, lawn green, lake blue, and lilac, respectively. The collinearity gene pairs were indicated by red lines.

Collinearity analyses among *M. lasiocarpa*, *Musa balbisiana*, *A. thaliana*, and *Oryza sativa* were conducted to investigate the evolutionary relationships among these model species, including dicotyledonous and monocotyledons. The interspecies collinearity analysis revealed that *MlWRKYs* exhibited syntenic relationships with genes on all chromosomes of *A. thaliana* (**Figure 3**). Notably, the number of collinear gene pairs between *M. lasiocarpa* and *O. sativa* exceeded that between *M. lasiocarpa* and *A. thaliana*, likely due to both *M. lasiocarpa* and *O. sativa* being monocotyledons. Similarly, a higher number of syntenic gene pairs were observed between *M. lasiocarpa* and *M. balbisiana*, attributable to their closer evolutionary relationship within the Musaceae family.

### 3.4 Integration of multi-omics approaches to identify candidate MlWRKYs for regulating PhPNs biosynthesis

**Figure 4:**
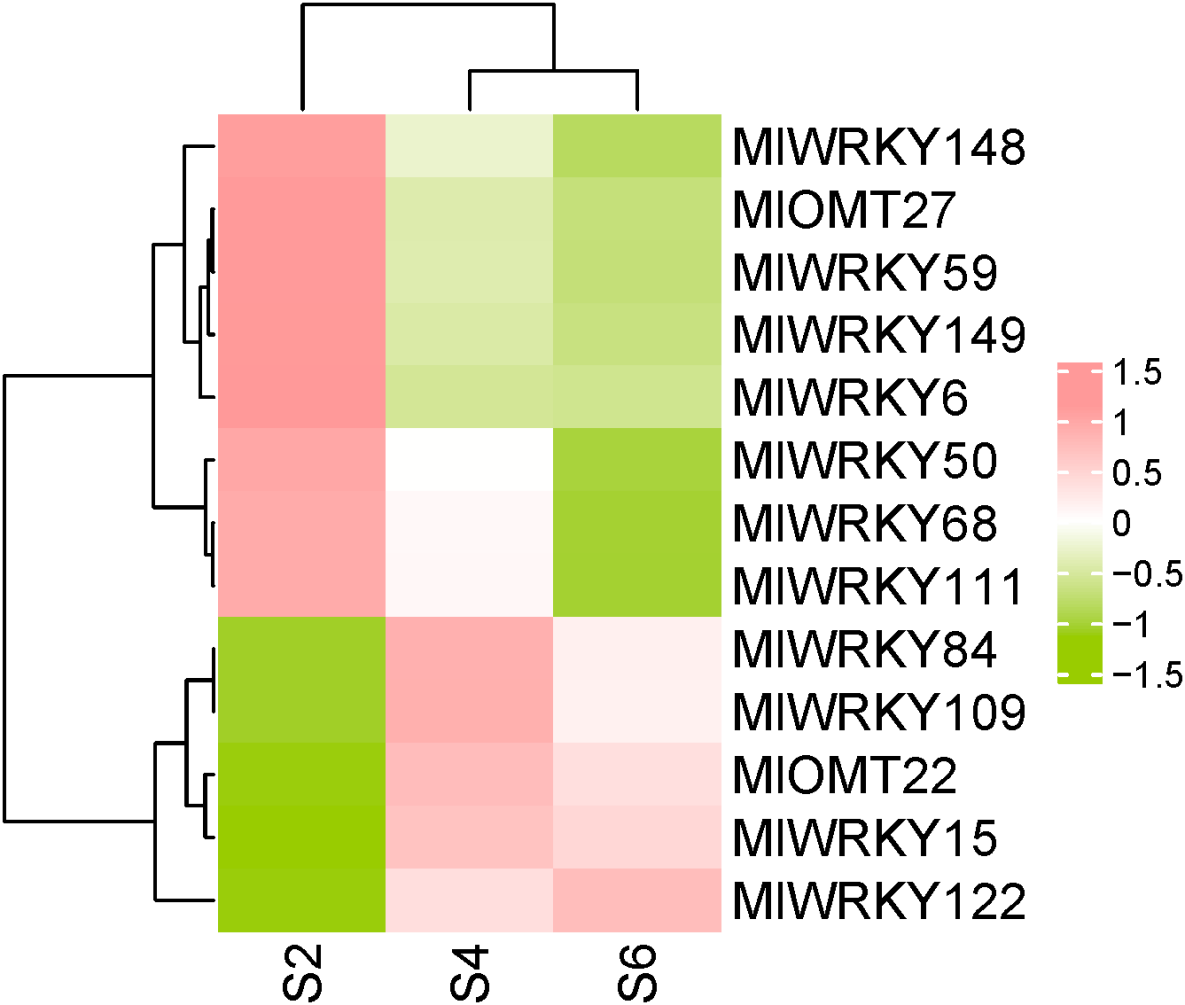
Heat map representing the expression levels of *MlWRKY* and *MlOMT* genes. The heat map illustrating the expression profiles of *MlWRKY* and *MlOMT* genes across in *M. lasiocarpa* seeds during different developmental stages, measured by FPKM. Eleven *MlWRKY* genes (*MlWRKY6*, *MlWRKY15*, *MlWRKY50*, *MlWRKY59*, *MlWRKY68*, *MlWRKY84*, *MlWRKY109*, *MlWRKY111*, *MlWRKY122*, *MlWRKY148*, and *MlWRKY149*) had an FPKM value exceeding 20 in least one of the tested samples. The color bar indicates gene expression levels, with expression increasing from green to red.

We previously reported PhPNs content in yellow seed (stage S2), brown seed (S4), and block seed (S6) of *M. lasiocarpa*. The results demonstrated a gradual increase in PhPN content, following the order S2 < S4 < S6 (Zhao et al., 2024). To investigate the expression patterns of MlWRKYs in *M. lasiocarpa* seeds across different developmental stages, RNA-Seq data from previous studies (accession PRJNA1009687) were analyzed. A heat map generated using FPKM values illustrated the expression profiles of *MlWRKYs* in seeds at S2, S4, and S6 stages (**Figure 4**). The majority of genes exhibited either no expression or low expression levels in the three developmental stages of *M. lasiocarpa* seeds. Only 11 out of 158 *MlWRKY* genes had an FPKM value exceeding 20 in least one of the tested samples. Among these 11 genes, *MlWRKY15*, *MlWRKY84*, *MlWRKY109*, and *MlWRKY122* showed expression patterns consistent with the growth trend of PhPNs content. While *MlWRKY6*, *MlWRKY50*, *MlWRKY59*, *MlWRKY148*, and *MlWRKY149* showed opposite expression pattern with the growth trend of PhPNs content. Previous research has demonstrated that *CsWRKY57like* from *Camellia sinensis* influences the biosynthesis of methylated epigallocatechin gallate (EGCG) by regulating the *CCoAOMT* gene (Luo et al., 2022). A homology search using the amino acid sequence of CsWRKY57like as a query against MlWRKYs revealed higher similarity with *MlWRKY15*, *MlWRKY111*, and *MlWRKY122*. Based on these findings, we hypothesize that *MlWRKY15*, *MlWRKY111*, and *MlWRKY122* are likely to regulate PhPNs biosynthesis through the modulation of *MlOMT* genes expression. Furthermore, our previous studies demonstrated that two *MlOMT* genes, *Ml04G2958* (*MlOMT22*), and *Ml08G0855* (*MlOMT27*), are involved in PhPNs biosynthesis (Zhao et al., 2024). The expression pattern of *MlOMT22* was consistent with those of *MlWRKY15* and *MlWRKY122*, whereas the expression pattern of *MlOMT27* was consistent with that of *MlWRKY111* (**Figure 4**). Furthermore, the identification of W-box motifs in the promoters of *MlOMT22* and *MlOMT27* indicates their potential regulation by MlWRKY15, MlWRKY111, and MlWRKY122, providing insights into the transcriptional network underlying secondary metabolism in *M. lasiocarpa*.

### 3.5 Expression pattern of *MlWRKYs* in *M. lasiocarpa* during different tissues

**Figure 5:**
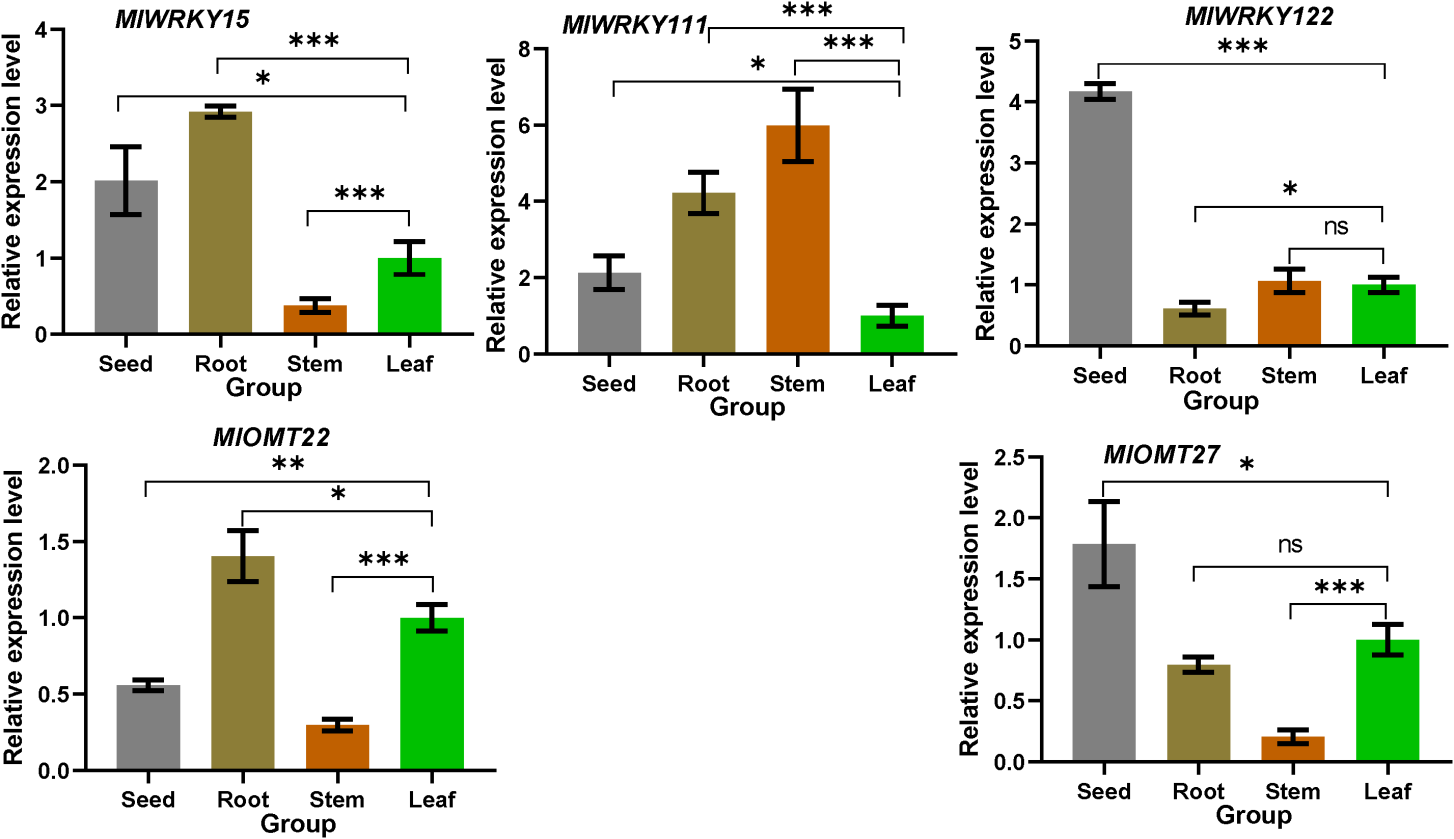
Expression patterns of three selected MlWRKY genes and their candidate target MlOMT genes in different tissues of *M. lasiocarpa*. All experiments were performed in triplicate, with relative expression levels normalized to the *EF-α* gene of *M. lasiocarpa*. Data are presented as mean ± SD. *, **, and *** denote that the genes from other tissues exhibit significant differences compared to leaf (with *P* values less than 0.05, 0.01, and 0.001 respectively). “ns” indicates no significant difference (P ≥ 0.05).

We analyzed the transcriptional levels of *MlWRKY* genes, *MlWRKY15*, *MlWRKY111*, and *MlWRKY122* and their candidate target genes *MlOMT22* and *MlOMT27* in four different tissues of *M. lasiocarpa* by qRT-PCR. It demonstrated that each gene could be detected in all four tested tissues (**Figure 5**). *MlWRKY15* and *MlOMT22* are predominantly expressed in the root, exhibiting similar expression patterns across different tissues. This suggests that *MlWRKY15* may positively regulate *MlOMT22* expression in various tissues. Similarly, *MlWRKY122* and *MlOMT27* are primarily expressed in seeds (black seeds), with consistent expression patterns across different tissues, indicating that *MlWRKY122* is likely to regulate *MlOMT27* expression in these tissues. Moreover, the expression pattern of *MlWRKY111* is inversely correlated with that of *MlOMT27*, suggesting a potential negative regulatory role of *MlWRKY111* on *MlOMT27*. However, further experimental validation is required to confirm these specific regulatory mechanisms.

## 4 Discussion

WRKY transcription factors exert essential regulatory functions in the biosynthesis of plant secondary metabolites (Yang et al., 2012; Kumar et al., 2023). They modulate the equilibrium between plant defense responses and metabolic pathways via transcriptional and signaling regulatory mechanisms. As our understanding of WRKY transcription factors deepens, their regulatory roles in plant secondary metabolism and their potential for industrial applications—such as metabolic engineering and biopharmaceutical production—will be increasingly clarified. Future studies should focus on the interactions of WRKY transcription factors with other transcription factors and signaling pathways, as well as their applications in plant metabolic engineering. For instance, transgenic or gene editing technologies can be employed to enhance WRKY transcription factor expression, thereby improving the yield and quality of plant secondary metabolites (Das et al., 2024). Banana is one of the most important fruits globally, yet its production faces significant threats from various diseases, particularly those caused by fungi, bacteria, and viruses (Drenth and Kema, 2021). While traditional chemical pesticides are widely employed to protect bananas from these pathogens, concerns about their potential environmental and health risks have spurred research into natural, efficient, and less toxic alternatives (Ferreyra-Suarez et al., 2024). PhPN, a type of natural secondary metabolite, plays a crucial role in plant self-protection, demonstrating significant biological activity in disease resistance, antibacterial, and antifungal properties (Krishnamurthy et al., 2023). Additionally, ketenes exhibit substantial potential in antioxidant, anti-inflammatory, antibacterial, and anticancer activities, with broad applications in drug development, food, fragrance, and cosmetics industries. PhPN, derived from phenylpropanoid compounds, typically features a phenyl-ketene structure, comprising a benzene ring and an unsaturated ketene moiety. This structural configuration enables PhPN to engage in diverse biochemical reactions and interact with various biomolecules. Notably, PhPN content in bananas is very low; it primarily exists in certain plants of the Musaceae family, such as *M. lasiocarpa*, which is rich in PhPN and serves as an ideal material for studying its biosynthesis and regulation (Zhao et al., 2024).

Plants regulate their responses to various stresses through transcription factors such as WRKY (Liu et al., 2020) (Li et al., 2024; Ma et al., 2024a), with an important mechanism being the regulation of secondary metabolite biosynthesis (Jiang et al., 2017). In recent years, the role of WRKY transcription factors in regulating secondary metabolite biosynthesis has garnered significant attention (Li et al., 2025; Zhao et al., 2025b). WRKY transcription factors recognize and bind to the W-box cis-element (TTGACC/T) in the promoters of target genes. For instance, SlWRKY35 from tomatoes directly activates the expression of the *SlDXS1* gene, thereby enhancing carotenoid biosynthesis and accumulation (Yuan et al., 2022). Conversely, ElWRKY48 negatively regulates ingenol biosynthesis by modulating the expression of genes involved in diterpenoid biosynthesis (Zhao et al., 2025a). We identified similar binding motifs (W-box) in the promoters of MlOMT22 and MlOMT27 genes in *M. lasiocarpa*, suggesting a conserved regulatory mechanism. Our co-expression analysis revealed that *MlWRKY15*, *MlWRKY111* and *MlWRKY122* similar expression patterns with *MlOMT22* and *MlOMT27*, implying potential protein-protein interactions that may synergistically activate PhPN biosynthetic genes. However, it remains unclear how MlWRKYs regulate the biosynthesis of PhPNs by modulating the expression of *MlOMT* genes, which will be the focus of our future research. We selected MlWRKY15, MlWRKY111, and MlWRKY122 for tissue expression analysis based on the following evidence: 1) Expression Patterns: As shown in **Figure 4**, *MlWRKY15*, *MlWRKY111*, and *MlWRKY122* exhibited expression patterns consistent with the growth trend of PhPNs content in *M. lasiocarpa* seeds (S2 < S4 < S6). This correlation suggests a potential regulatory role in PhPNs biosynthesis. 2) Homology Analysis: A homology search using the amino acid sequence of *CsWRKY57like* (a known regulator of methylated EGCG biosynthesis in *Camellia sinensis*) revealed higher similarity with *MlWRKY15*, *MlWRKY111*, and *MlWRKY122*. This finding further supports their potential involvement in the regulation of methylated compounds, such as PhPNs. 3) Functional Relevance: Previous studies have demonstrated that WRKY transcription factors often regulate secondary metabolite biosynthesis by modulating the expression of key enzymes (e.g., *O*-methyltransferases). Given the homology and expression patterns, we hypothesize that MlWRKY15, MlWRKY111, and MlWRKY122 may regulate PhPNs biosynthesis through the modulation of MlOMT genes.

In this study, we performed the first comprehensive analysis of the WRKY gene family in *M. lasiocarpa*. In sum, 158 *MlWRKY* genes were identified in the genome, showing an uneven distribution across all nine chromosomes. We systematically examined the evolution, chromosomal localization, protein-protein interactions, and expression patterns of these *MlWRKY* genes. Specifically, *MIWRKY15*, *MIWRKY111*, and *MIWRKY122* were pinpointed as candidate genes potentially involved in the regulation of PhPN biosynthesis. These genes represent promising targets for future functional studies aimed at elucidating their roles in PhPN biosynthesis in *M. lasiocarpa*. This study lays a solid foundation for future research into the functions of *MlWRKY* genes and the molecular mechanisms governing PhPN biosynthesis. While this study has identified potential WRKY genes that may regulate PhPN biosynthesis, the specific regulatory mechanisms remain to be elucidated, which will be a focus of our subsequent research. Overall, this is the first identification of the WRKY gene family in *M. lasiocarpa*, providing a basis for understanding the biosynthetic mechanism of PhPNs and offering insights into other potential functions of WRKY genes in plants.

## 5 Conclusion

In summary, this study represents the first comprehensive analysis of the WRKY gene family in *M. lasiocarpa*, identifying 158 *MlWRKY* genes unevenly distributed across nine chromosomes. We characterized their evolutionary relationships, chromosomal distribution, protein-protein interactions, and expression patterns. *MIWRKY15*, *MIWRKY111*, *MIWRKY122* were identified as candidate genes potentially involved in regulating PhPNs biosynthesis by integration of multi-omics approaches. These genes serve as promising candidates for future functional studies aimed at elucidating their roles in PhPNs biosynthesis regulation in *M. lasiocarpa*. This research provides a foundation for further investigation into the functions of *MIWRKY* genes and the molecular mechanisms underlying PhPNs biosynthesis.

## Data Availability

The datasets presented in this study can be found in online repositories. The names of the repository/repositories and accession number(s) can be found in the article/**Supplementary Material**.
