# Supplementary material for: Comprehensive characterization of the WRKY gene family and their potential roles in regulation phenylphenalenone biosynthesis in Musella lasiocarpa
Source: Front Plant Sci. 2025 Mar 12;16:1570758. doi: 10.3389/fpls.2025.1570758 (PMC11936918; doi:10.3389/fpls.2025.1570758)
Supplement: Supplementary file 1 [file DataSheet1.docx]

Table S1 The primers utilized for qRT-PCR in this study.

| Name | Sequence (5'-3') | Product length (bp) |
| --- | --- | --- |
| MlOMT22-F | TCAACTACGACCTCCCTC | 148 |
| MlOMT22-R | GCACAATGCTCATCACTC |  |
| MlOMT27-F | ATCAACGCCAAGAACACC | 312 |
| MlOMT27-R | CCTCACCAGATCCAGCAG |  |
| MlWRKY15-F | TTGGCAGAAACAGGGGAT | 308 |
| MlWRKY15-R | TGGCACTGACTTGGGGAC |  |
| MlWRKY111-F | GCCTCCTTCCTCTTCCCC | 270 |
| MlWRKY111-R | CTTCTGCCCGTACTTGCG |  |
| MlWRKY122-F | TGACCCTTCTAACTCCCT | 304 |
| MlWRKY122-R | CTTCTGACCATACTTGCG |  |
| EF-α-F | CATTCAAAAACCACTACCCATC | 138 |
| EF-α-R | CTCCATTCATTTCAGTCATCGC |  |

Note, the *EF-α* gene (Elongation Factor-1α) from *M. lasiocarpa* serves as the internal reference gene for q-PCR analysis.


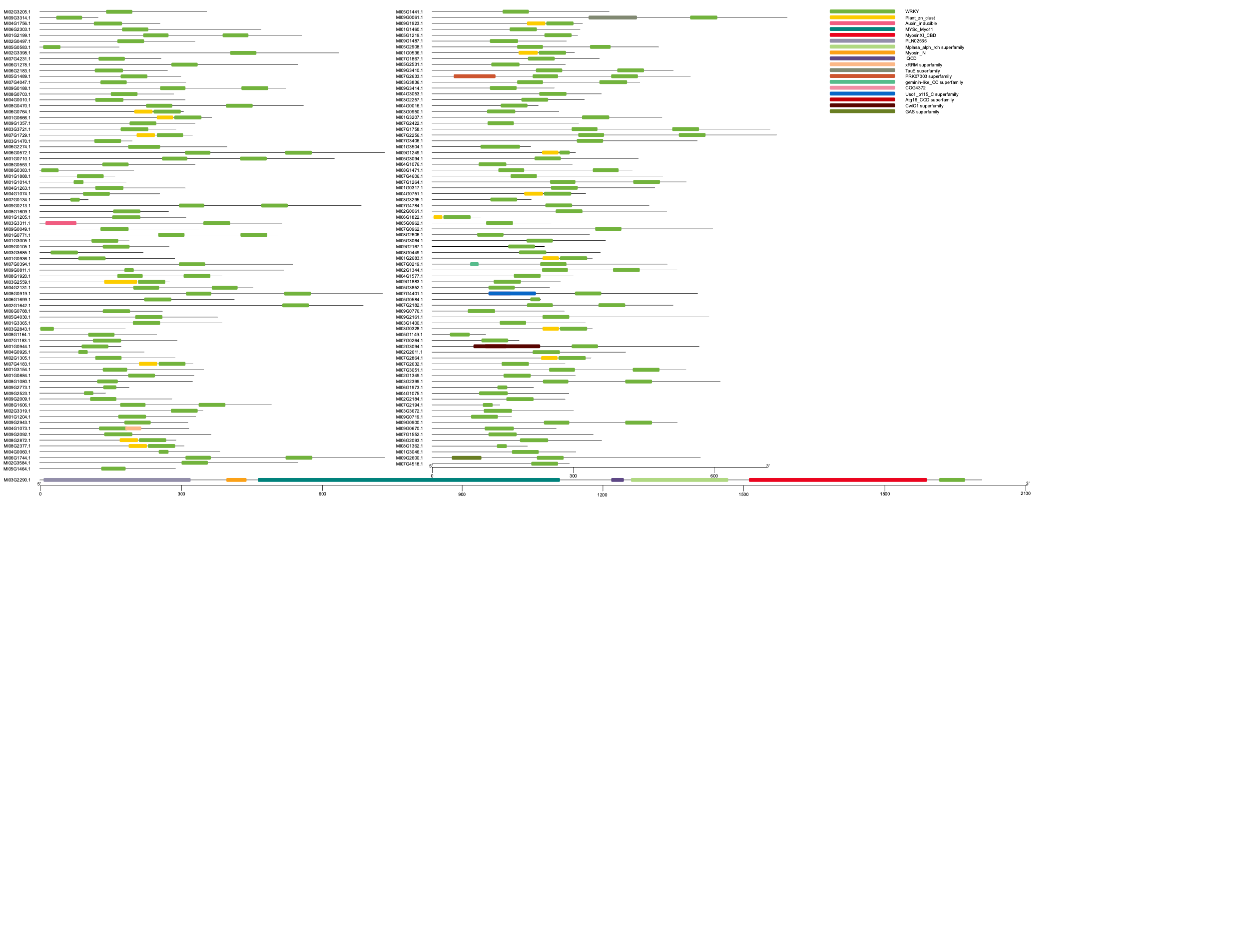


Fig. S1. Analysis of conserved domain compositions in MlWRKY proteins. All of 158 MlWRKY genes were found to possess at least one WRKY conserved domain.
